# Comparison of Outpatient Satisfaction Survey Scores for Asian Physicians and Non-Hispanic White Physicians

**DOI:** 10.1001/jamanetworkopen.2019.0027

**Published:** 2019-02-22

**Authors:** Luis C. Garcia, Sukyung Chung, Lily Liao, Jonathan Altamirano, Magali Fassiotto, Bonnie Maldonado, Paul Heidenreich, Latha Palaniappan

**Affiliations:** 1 Office of Faculty Development and Diversity, Stanford University School of Medicine, Stanford, California; 2 Palo Alto Medical Foundation Research Institute, Palo Alto, California; 3 Department of Medicine, Stanford University School of Medicine, Stanford, California; 4 Columbia University Vagelos College of Physicians and Surgeons, New York, New York; 5 Department of Pediatrics, Stanford University School of Medicine, Stanford, California

## Abstract

**Importance:**

Patient satisfaction scores are used to inform decisions about physician compensation, and there remains a lack of consensus regarding the need to adjust scores for patient race/ethnicity. Previous research suggests that patients prefer physicians of the same race/ethnicity as themselves and that Asian patients provide lower satisfaction scores than non-Hispanic white patients.

**Objective:**

To examine whether Asian physicians receive less favorable patient satisfaction scores relative to non-Hispanic white physicians.

**Design, Setting, and Participants:**

This population-based survey study used data from Press Ganey Outpatient Medical Practice Surveys collected from December 1, 2010, to November 30, 2014, which included 149 775 patient survey responses for 962 physicians. Every month, 5 patients per physician were randomly selected to complete a satisfaction survey after an outpatient visit. Hierarchical multivariable logistic regression was used to examine the association between Asian race/ethnicity of the physician and racial/ethnic concordance of the patient with the probability of receiving the highest score on the survey item rating the likelihood to recommend the physician. Statistical analysis was performed from April 2 to August 27, 2018.

**Exposures:**

Physician characteristics included race/ethnicity, sex, years in practice, and proportion of Asian patient responders. Patient characteristics included race/ethnicity, sex, age, and language spoken.

**Main Outcomes and Measures:**

The highest score (a score of 5 on a 1-5 Likert scale, where 1 indicates very poor and 5 indicates very good) on the survey item rating the likelihood to recommend the physician on the Press Ganey Outpatient Medical Practice Survey.

**Results:**

Of the 962 physicians in this study, 515 (53.5%) were women; physicians had a mean (SD) of 19.9 (9.1) years of experience since graduating medical school; 573 (59.6%) were white, and 350 (36.4%) were Asian. In unadjusted analyses, the odds of receiving the highest score on the survey item rating the likelihood to recommend the physician were lower for Asian physicians compared with non-Hispanic white physicians (odds ratio, 0.78; 95% CI, 0.72-0.84; *P* < .001). This association was not significant after adjusting for patient characteristics, including patient race/ethnicity. However, Asian patients were less likely to give the highest scores relative to non-Hispanic white patients (odds ratio, 0.56; 95% CI, 0.54-0.58; *P* < .001), regardless of physician race/ethnicity.

**Conclusions and Relevance:**

This study suggests that Asian physicians may be more likely to receive lower patient satisfaction scores because they serve a greater proportion of Asian patients. Patient satisfaction scores should be adjusted for patient race/ethnicity.

## Key Points

**Question:**

Do Asian physicians receive lower patient satisfaction scores relative to non-Hispanic white physicians?

**Findings:**

In this population-based survey study using data collected from 149 775 patient satisfaction surveys, Asian physicians received lower patient satisfaction scores relative to non-Hispanic white physicians. Asian physicians served a greater proportion of Asian patients, who were more likely to give lower satisfaction scores than non-Asian patients.

**Meaning:**

Health organizations using patient satisfaction scores for physician compensation and promotion should consider statistical adjustments for patient race/ethnicity.

## Teaser

This population-based survey study uses data from 149 775 patient surveys to compare patient satisfaction scores for Asian physicians relative to non-Hispanic white physicians.

## Introduction

Measures of patient satisfaction have become increasingly important during the last decade. By affecting hospital reimbursements and physician compensation in the form of performance bonuses, measures of patient satisfaction are associated with financial incentives to monitor and improve the quality of health care services and delivery.^1,2,3^

Furthermore, a growing body of literature has demonstrated that immutable, patient-level characteristics, such as age, overall health status, educational level, and race/ethnicity, serve as important determinants of patient-reported satisfaction.^4,5,6,7,8,9,10^ To avoid possible measurement bias, health organizations adjust patient satisfaction scores for various characteristics, including age, sex, and health status.^11,12^ For example, health organizations that use the Consumer Assessment of Healthcare Providers and Systems (CAHPS) survey to assess patient-reported satisfaction commonly adjust for a patient’s general and mental health status and educational level.^5^ However, the association between race/ethnicity and patient satisfaction scores—and the consequent need to make statistical adjustments for patient race/ethnicity—remains uncertain. Many previous studies have examined differences in the patient satisfaction scores of black or African American and Hispanic patients relative to non-Hispanic white patients (hereafter simply referred to as white patients).^13,14,15,16^ Fewer studies have investigated the association between Asian race/ethnicity of patients and patient satisfaction scores, and those that have were conducted outside of the United States, limited by small samples, and/or constrained by the use of patient satisfaction survey instruments with limited generalizability.^17,18,19,20,21,22,23^ The research that is currently available suggests that Asian patients assign lower patient satisfaction scores relative to other racial/ethnic groups.^17,18,19,20,21,22,23^

The Press Ganey Outpatient Medical Practice Survey is a validated instrument used in most outpatient practices in the United States to measure patient satisfaction and inform physician evaluations and compensation decisions.^24,25^ In a recent study, we demonstrated that Asian patients were significantly less likely to provide the highest scores on the Press Ganey survey when evaluating physicians, even after controlling for a patient’s sex and age.^26^ Because previous research has demonstrated that patients may be more likely to select physicians of the same race/ethnicity as themselves,^27^ the purpose of the present investigation was not only to examine whether Asian physicians receive less favorable patient satisfaction scores relative to non-Hispanic white physicians (hereafter simply referred to as white physicians) but also to consider whether these lower scores are mediated by the proportion of Asian individuals composing physicians’ patient panels.

## Methods

### Study Design and Setting

This was an observational, population-based survey study using the results of Press Ganey patient satisfaction surveys. Survey data were collected from December 1, 2010, to November 30, 2014, at community-based outpatient clinics that are part of a not-for-profit health organization in northern California. Approval for this study was provided by the institutional review board at the Palo Alto Medical Foundation Research Institute. The need for informed consent was waived because the data were deidentified. This study followed the Strengthening the Reporting of Observational Studies in Epidemiology (STROBE) reporting guideline.

### Survey Sampling

After outpatient office visits, surveys were administered by the health care system to 5 randomly selected patients per physician per month. Patients with multiple visits were selected to complete the satisfaction survey only once per 90 days. Data were obtained from 149 775 patient survey responses for 962 physicians. During the study period, the response rate was 17.5%. The response rate was calculated according to standard definitions established by the American Association for Public Opinion Research (AAPOR) reporting guideline.^28^

### Survey Instrument

The Press Ganey Outpatient Medical Practice Survey is the most commonly used assessment tool for patient satisfaction in US ambulatory centers.^24,25^ The survey consists of 6 domains (comprising 2-10 questions each), including nurse or assistant, care provider, personal issues, overall assessment, access, and moving through the visit (in this study, data were obtained only from encounters involving a physician, so the care provider survey domain is hereafter referred to as the physician domain). Individual survey items are scored using a 5-point Likert scale, with responses ranging from very poor (a score of 1) to very good (a score of 5). Patient demographic data collected through the survey include self-reported age, sex, and race/ethnicity.

### Study Outcome

When data are collected using the Press Ganey Outpatient Medical Practice Survey, responses are heavily skewed toward the highest possible rating, and therefore responses are frequently classified dichotomously.^29^ Scores of 5 serve as the most favorable score and are compared with all other possible responses grouped together (scores of 1-4).^29^ Patients’ responses when answering the “likelihood of your recommending this care provider to others” survey item are particularly important. Many health care organizations across the country—including the site of data collection—use the “likelihood to recommend” (LTR) survey item of the physician domain to inform physician assessments, monetary performance bonuses, and rankings.^30,31,32,33,34^ Therefore, the decision to use the most favorable LTR scores as the outcome of interest in this study was made given the use of this survey item in physician ranking and compensation decisions by health care organizations plus consensus among the research team that LTR was most closely associated in meaning with the overall physician domain.

### Statistical Analysis

Statistical analysis was performed from April 2 to August 27, 2018. Data collected using Press Ganey surveys were linked to patient medical records and deidentified for analysis. Bivariate associations between the race/ethnicity of the physicians and the percentage of Asian patients composing physicians’ patient panels were assessed using 1-way analysis of variance. Logistic regression with physician-level random effects was used to examine the association between the race/ethnicity of the physician and the odds of receiving the most favorable LTR scores while adjusting for clustering of patients by physician. Individual survey responses served as the unit of analysis. The association between the race/ethnicity of the physician and the most favorable LTR scores was first modeled using univariate logistic regression. In subsequent multivariable models, statistical adjustments were made for physician and patient characteristics associated with patient satisfaction scores, including patient and physician sex, patient age, physician experience, and clinical department. A final multivariable model was used to assess the association of patient-physician racial/ethnic concordance on the odds of receiving the most favorable LTR scores. Hispanic physicians and black or African American physicians were excluded from the concordance analysis owing to limited sample sizes. All *P* values were from 2-tailed tests, and the results were deemed statistically significant at *P* < .05.

Patient age was categorized into the following 3 levels: 18 to 44 years, 45 to 64 years, and 65 years or older. Physician experience was classified based on the number of years since completing medical school as follows: less than 15 years, 15 to 24 years, and more than 24 years. Race/ethnicity was self-reported and classified as white, black or African American, Asian, and Hispanic. Data from respondents who selected “mixed” or “other” race/ethnicity or whose race/ethnicity was unknown were excluded from analysis. The accuracy of self-reported patient race/ethnicity has been demonstrated previously.^35^ Self-reported patient primary language was classified as English or non-English. The proportion of Asian patient respondents served as a proxy for the proportion of Asian patients comprising a physician’s patient panel. Asian patient race/ethnicity included disaggregated data, allowing for concordant pairs to be defined using Asian subgroups (Asian Indian, Chinese, Filipino, Japanese, Vietnamese, and Korean). Data were analyzed using Stata, version 13.1 (StataCorp).

## Results

There were a total of 962 physicians in the study. They were predominantly female (515 [53.5%]) and white (573 [59.6%]; Table 1). Asian physicians comprised more than one-third of the overall sample (350 [36.4%]). Among Asian physicians, 165 (47.1%) were Chinese, and 99 (28.3%) were Indian. Most physicians (610 [63.4%]) had more than 15 years of clinical experience beyond medical school. The mean (SD) years of experience since graduating medical school was 19.9 (9.1) years. In terms of mean (SD) percentages, Asian patients composed 20.9% (20.8%) of white physicians’ patient panels; in contrast, Asian patients composed 43.9% (40.6%) of Asian physicians’ patient panels (*P* < .001). In terms of mean (SD) percentages, Asian patients composed 25.0% (22.5%) of Hispanic physicians’ patient panels and 25.8% (29.0%) of black or African American physicians’ patient panels; these percentages were not significantly different relative to white physicians. The unadjusted odds of receiving the most favorable LTR score were 22% lower for Asian physicians relative to white physicians (odds ratio [OR], 0.78; 95% CI, 0.72-0.84; *P* < .001; Table 2, model 1).

**Table 1.** Data on Patient Visits by Physician Demographic Characteristics

Physician Characteristic	No. (%)	
	Physicians (N = 962)	Patient Survey Responses (N = 149 775)
Race/ethnicity		
Non-Hispanic white	573 (59.6)	91 042 (60.8)
Asian	350 (36.4)	52 805 (35.3)
Hispanic	23 (2.4)	3986 (2.7)
Black or African American	16 (1.7)	1942 (1.3)
Sex		
Male	447 (46.5)	73 014 (48.7)
Female	515 (53.5)	76 761 (51.3)
Years in practice		
<15	352 (36.6)	55 291 (36.9)
15-24	364 (37.8)	49 197 (32.8)
>24	246 (25.6)	45 287 (30.2)

**Table 2.** Odds of Receiving the Most Favorable Likelihood to Recommend Physician Score, by Physician and Patient Characteristics

Variable	Model 1		Model 2		Model 3	
	OR (95% CI)	*P* Value	OR (95% CI)	*P* Value	OR (95% CI)	*P* Value
**Physician**						
Race/ethnicity						
Non-Hispanic white	1 [Reference]	NA	1 [Reference]	NA	1 [Reference]	NA
Asian	0.78 (0.72-0.84)	<.001	0.94 (0.86-1.02)	.15	0.93 (0.86-1.02)	.11
Hispanic	0.93 (0.75-1.16)	.54	1.00 (0.80-1.24)	.97	1.02 (0.82-1.26)	.88
Black or African American	0.81 (0.62-1.07)	.13	0.91 (0.69-1.19)	.48	0.93 (0.71-1.21)	.57
Sex						
Male	NA	NA	1 [Reference]	NA	1 [Reference]	NA
Female	NA	NA	1.03 (0.95-1.11)	.47	1.04 (0.96-1.12)	.31
Years in practice						
<15	NA	NA	1 [Reference]	NA	1 [Reference]	NA
15-24	NA	NA	1.19 (1.12-1.26)	<.001	1.15 (1.09-1.22)	<.001
>24	NA	NA	1.21 (1.12-1.30)	<.001	1.15 (1.07-1.25)	<.001
% of Asian respondents			0.35 (0.25-0.48)	<.001	1.04 (0.75-1.44)	.81
**Patient**						
Race/ethnicity						
Non-Hispanic white	NA	NA	NA	NA	1 [Reference]	NA
Asian	NA	NA	NA	NA	0.56 (0.54-0.58)	<.001
Hispanic	NA	NA	NA	NA	0.93 (0.83-1.04)	.19
Black or African American	NA	NA	NA	NA	0.99 (0.94-1.05)	.74
Sex						
Male	NA	NA	NA	NA	1 [Reference]	NA
Female	NA	NA	NA		1.04 (1.01-1.07)	.02
Age, y						
18-44	NA	NA	NA	NA	1 [Reference]	NA
45-64	NA	NA	NA	NA	1.48 (1.42-1.53)	<.001
≥65	NA	NA	NA	NA	1.58 (1.52-1.65)	<.001
Language						
English	NA	NA	NA	NA	1 [Reference]	NA
Non-English	NA	NA	NA	NA	0.59 (0.56-0.62)	<.001

Abbreviations: NA, not applicable; OR, odds ratio.

### Adjustment for Physician Characteristics

After statistical adjustment for a physician’s sex and years of experience and the proportion of Asian patient respondents (Table 2, model 2), the odds of receiving the most favorable LTR score did not differ by physician race/ethnicity as observed in model 1. Having Asian patients decreased physicians’ odds of receiving the most favorable LTR score. When the proportion of a physician’s Asian patient respondents increased by 1%, the physician’s odds of receiving the most favorable LTR score was reduced by 65% (OR, 0.35; 95% CI, 0.25-0.48; *P* < .001). Physician experience was positively and significantly associated with the odds of receiving the most favorable LTR scores.

### Adjustment for Physician and Patient Characteristics

After adjustment for physician and patient characteristics (Table 2, model 3), the association between the Asian race/ethnicity of the physician and receiving the most favorable LTR score remained nonsignificant (OR, 0.93; 95% CI, 0.86-1.02; *P* = .11). Asian patients were less likely to give the most favorable LTR score compared with white patients (OR, 0.56; 95% CI, 0.54-0.58; *P* < .001). Physician experience and patient age were positively and significantly associated with the most favorable LTR scores, whereas patients whose primary language was not English were significantly less likely to give the most favorable LTR scores.

### Racial Concordance

In a final multivariable model, racially/ethnically concordant and discordant patient-physician dyads were compared with concordant, white physician–white patient pairs (Table 3). Regardless of racial/ethnic concordance and relative to white physician–white patient pairs, the odds of a most favorable LTR score were lower for all dyads including an Asian patient. Physician experience and patient age remained significantly associated with greater odds of receiving the most favorable LTR scores, and non-English as the primary language remained inversely and significantly associated with the odds of giving the most favorable LTR scores.

**Table 3.** Odds of Receiving the Most Favorable Likelihood to Recommend Physician Score, by Patient-Physician Racial/Ethnic Concordance

Variable	OR (95% CI)	*P* Value
Non-Hispanic white physician		
Non-Hispanic white patient	1 [Reference]	NA
Asian patient	0.52 (0.49-0.54)	<.001
Hispanic patient	0.95 (0.88-1.02)	.13
Black or African American patient	0.88 (0.76-1.02)	.09
Asian physician		
Asian patient concordant	0.53 (0.47-0.59)	<.001
Asian patient discordant	0.55 (0.50-0.61)	<.001
Non-Hispanic white patient	0.87 (0.80-0.96)	.003
Hispanic patient	0.92 (0.82-1.04)	.18
Black or African American patient	0.81 (0.66-0.98)	.03
Physician sex		
Male	1 [Reference]	NA
Female	1.05 (0.97-1.14)	.21
Years in practice		
<15	1 [Reference]	NA
15-24	1.15 (1.09-1.22)	<.001
>24	1.16 (1.07-1.25)	<.001
% of Asian respondents	1.05 (0.76-1.46)	.77
Patient sex		
Male	1 [Reference]	NA
Female	1.04 (1.00-1.07)	.03
Patient age, y		
18-44	1 [Reference]	NA
45-64	1.48 (1.42-1.55)	<.001
≥65	1.59 (1.53-1.66)	<.001
Patient language		
English	1 [Reference]	NA
Non-English	0.58 (0.55-0.62)	<.001

Abbreviations: NA, not applicable; OR, odds ratio.

## Discussion

We found that Asian physicians received lower patient satisfaction scores relative to white physicians. However, this finding was attenuated after statistical adjustment for physician and patient characteristics, including the percentage of Asian patients comprising physicians’ patient panels. Regardless of the physician’s race/ethnicity, Asian patients gave consistently and substantially lower patient satisfaction scores. For Asian physicians, in particular, the likelihood of receiving the most favorable LTR score was the lowest after encounters between racially/ethnically concordant Asian physicians and Asian patients.

In addition, both Asian and white physicians received lower LTR scores from Hispanic patients and black or African American patients. Although these associations were only statistically significant between Asian physician–black or African American patient dyads, these findings are consistent with the previous literature on racial/ethnic concordance between physicians and patients, which has demonstrated that patient satisfaction is greater between racially concordant black or African American physician–patient pairs and Hispanic physician–patient pairs.^13,14,15,16^ Thus, whereas the satisfaction scores of black or African American physicians and Hispanic physicians may be improved by interactions with racially concordant patients, the satisfaction scores of Asian physicians are doubly penalized by the greater proportion of Asian patients whom they serve and their discordant racial/ethnic patient interactions. This finding also aligns with the results of a study in which we demonstrated that Asian patients were less likely than white patients to give the most favorable LTR scores even after adjusting for known confounders.^26^

Our finding that lower LTR scores given by Asian patients explains the lower LTR scores of Asian physicians should be considered with past work demonstrating that Asian patients are more likely to select midpoints, rather than extremes, when completing Likert-type^36^ and CAHPS^37^ surveys, which may consequently diminish the likelihood that Asian patients will provide the most favorable ratings. In another study, Asian patients in an outpatient center perceived longer wait times and reported lower patient satisfaction than other racial/ethnic minority groups, even though electronic health record data revealed no significant differences in mean wait times.^38^ In addition, Saha and Hickam^22^ demonstrated that more dissatisfied Asian patients were not more likely to change physicians, which suggests that lower patient satisfaction scores may less accurately capture the experience of Asian patients. Together, these findings provide evidence to suggest that cultural response bias, rather than true differences in quality of care, may account for lower patient satisfaction scores received by Asian physicians and given by Asian patients. Finally, these results are particularly relevant given the large proportion of Asian patients residing in California. Previous literature suggests that CAHPS scores are lower in California relative to the rest of the nation, although this result is no longer observed after multivariable adjustment for possible confounders, including patient race/ethnicity.^39^

The results of this study should also be interpreted in the context of previous literature suggesting differences in the experiences of Asian physicians compared with those of nonminority counterparts. For example, Nunez-Smith et al^40^ found that 45% of Asian physicians experienced racial/ethnic discrimination sometimes, often, or very often during their medical careers compared with 7% of white physicians. In a 2017 national survey of US physicians, 43% of Asian physicians reported hearing biased remarks about their race from patients compared with 11% of white physicians.^41^ Asian physicians are considered to be overrepresented in biomedicine, but they remain underrepresented as department chairs, medical school deans, and senior investigators.^42,43,44^ Taken together, the results of this and previous studies therefore suggest that Asian physicians experience challenges that are similar to challenges faced by their minority counterparts.

In practice, a physician’s sex is commonly considered in performance assessment, but race/ethnicity is not. Our study emphasizes the importance of statistical adjustments of a patient’s race/ethnicity because Asian race was associated much more strongly with lower LTR scores than was a physician’s sex.

### Limitations

This study has multiple limitations to consider. The data on patients and physicians used in this analysis were not paired with quality or clinical outcomes data, consequently limiting the ability to ascertain whether Asian physicians provided similar quality care as their white counterparts. Furthermore, the design of this study does not allow for causal inference. It is possible that the lower scores received by Asian physicians relative to their white counterparts are explained by confounders that were unaccounted for in this analysis. Although the observed response rate was comparable to previous published studies involving the Press Ganey Outpatient Medical Practice Survey,^45,46,47^ the possibility of nonresponse bias suggests that these results may have limited generalizability and representativeness when considering Asian physicians and patients in other contexts and geographic settings. There were too few black or African American physicians and Hispanic physicians for inclusion in the concordance analysis. Thus, future research in geographical settings outside of California (with lower proportions of Asian patients and including greater sample sizes of black or African American physicians and patients and Hispanic physicians and patients) are necessary to validate these results.

## Conclusions

In this study, Asian physicians received lower LTR scores than did other physicians. Asian physicians served a greater proportion of Asian patients, who were more likely to give lower satisfaction scores than non-Asian patients. The results of the present study therefore suggest that statistically adjusting patient satisfaction scores by patient race/ethnicity may help avoid unduly penalizing physicians who serve greater proportions of Asian patients.
